# *In Vitro* Immunomodulatory Effect of Food Supplement from* Aloe vera*

**DOI:** 10.1155/2019/5961742

**Published:** 2019-03-03

**Authors:** Zaira López, Antoni Femenia, Gabriela Núñez-Jinez, Michelle N. Salazar Zúñiga, M. Eduardo Cano, Teresa Espino, Peter Knauth

**Affiliations:** ^1^Cell Biology Laboratory, Centro Universitario de la Ciénega, Universidad de Guadalajara, Av. Universidad 1115, 47810 Ocotlán (Jal.), Mexico; ^2^Department of Chemistry, University of Balearic Islands, Ctra. Valldemossa km 7.5, 07122 Palma de Mallorca, Spain; ^3^Laboratorio de Biofísica, Centro Universitario de la Ciénega, Universidad de Guadalajara, Av. Universidad 1115, 47810 Ocotlán (Jal.), Mexico; ^4^Laboratorio de Química, Centro Universitario de la Ciénega, Universidad de Guadalajara, Av. Universidad 1115, 47810 Ocotlán (Jal.), Mexico

## Abstract

Food industries typically use* Aloe vera* as concentrated (100× to 200×) and dried powders in their final products. These powders are obtained by extrusion of* Aloe* inner leaf gel (ILG) or* Aloe* whole leaf (WLP); the juice is filtered through diatomaceous earth and activated carbon before spray drying at temperatures below 70 °C. In another process,* Aloe* inner leaf gel was dried at ~80 °C and mashed to a powder rich in high molecular weight fibres and soluble polysaccharides (ILF). In contrast to ILG and WLP, the ILF sample was cytotoxic for the human intestinal cell line Caco-2 (CC_50_ = 1 g/l), even at concentrations below the recommended dose for human consumption. At lower concentrations (250 mg/l) with LPS challenged macrophage-like THP-1 cells decreased by 40% the release of the anti-inflammatory cytokine IL-10, whereas the release of the proinflammatory cytokine IL-1*β* increased by 35% (compared to untreated but challenged macrophage-like THP-1 cells). Unexpectedly, under the same conditions, the less cytotoxic ILG and WLP, both samples with a lower fibre content, significantly increased (up to 2.4 times) the release of IL-10, while the concentration of IL-1*β* remained unaltered and of TNF*α* decreased by 35%. Even more interesting is that a treatment of the ILF sample with activated carbon reduced its cytotoxicity and increased the IL-10 release (3.1 times). Based on these results, we suggest applying an activated carbon treatment on* Aloe*-starting products, which have high fibre content and have received high temperature treatment, in order to reduce their cytotoxicity and improve their immunomodulatory properties.

## 1. Introduction

The biological activities of raw extracts from* Aloe vera* have been the object of a broad variety of studies. Among the most interesting effects are induction of apoptosis [1, 2], hepatoprotection [3], antioxidant [4, 5], antibacterial [6, 7], antidiabetic [8], antihyperglycemic [9, 10], and anti-inflammatory effects [11–14]. Such effects are attributed to more than 70 potentially bioactive compounds [15] present in the* Aloe vera* plant; however, these biological activities can often be attributed to a synergistic action of more than one constituent rather than to a single compound [16].* Aloe vera* leaves have a waxy cuticle followed by a single cell epidermis and a thin layer of chlorenchyma cells with vascular bundles, which produce a bitter tasting exudate called* Aloe* latex. The central part of the leaf consists of a thick watery inner parenchyma, called* Aloe* fillet, and the extruded fillet is known as* Aloe *gel. Most* Aloe vera*-containing food products are made with* Aloe* gel, because it lacks a series of secondary metabolites of the* Aloe* latex such as glycosylated anthrones (*e.g.*, aloin) and nonglycosylated anthraquinones (*e.g.*, aloe emodin), which exhibit several toxic effects [17, 18]. Therefore, products containing more than 10 ppm aloin are not considered as “generally recognized as safe" (GRAS) by the US Food and Drug Administration (FDA) [19]. The dry mass of* Aloe* gel is mainly composed of crude fibres (35.5%), soluble saccharides (26.8%), ashes (23.6%), proteins (8.9%), and lipids (5.1%). The crude fibres consist of 72.2% polysaccharides, which in turn are composed mainly of mannose (52.8%), glucose (26.7%), uronic acids (13%), xylose (1.4%), and arabinose (1.2%). In fresh* Aloe* gel the soluble acemannan, an acetylated polysaccharide composed of 77%  *β*-1,4-linked-mannosyl and 11%  *β*-1,4-linked glycosyl residues with 0.7% terminal galactose, can be found; additional branching points are mainly at C_3_ with 1.7% mannosyl and at C_6_ with 2.5% glycosyl and 1.6% mannosyl residues achieving an average molecular weight of 30-40 kDa [20].

It is believed that the polysaccharides present in the* Aloe* gel are responsible for the immunomodulatory effects, since they can alter several interactions among cells of the immune system. When cells such as mouse RAW264.7 macrophages are exposed only to* Aloe vera* polysaccharides, the proinflammatory cytokine release (IL-1*β*, IL-6, IL-8, and TNF*α*) increases in a dose-dependent manner [21, 22] and this activity could be attributed to the fraction with a molecular weight of 5-400 kDa [22]. This effect may be explained by the fact that long chain acemannans (> 500 kDa at 50 *μ*g/ml) are able to activate a NF*κ*B-reporter system in nondifferentiated human THP-1 monocytes to a similar amount as 10 *μ*g/ml LPS [23]. As expected, when human peripheral blood macrophages are challenged with LPS, a proinflammatory cytokine release (IL-1*β*, IL-6, IL-8, and TNF*α*) is stimulated; although, when the cells are additionally exposed to* Aloe vera* polysaccharides, anti-inflammatory effects could be observed, the proinflammatory cytokine release was inhibited [12, 13], which may be explained by an inhibition of the P2X7-receptor for exogenous ATP, which in turn inhibits the expression of the NLRP3-inflammosome. Additionally, the phosphorylation of I*κ*B, which inhibits proinflammatory NF*κ*B-signalling, is also inhibited [13]. And, furthermore, in human gingival fibroblasts* Aloe vera* polysaccharides are able to activate the proinflammatory TLR5-signalling [24].

As the commercialization of* Aloe vera* products increases, concerns arise on varying product quality even when those are certified by the International Aloe Science Council (IASC) [25]. Starting products for foodstuff from* Aloe vera* are usually commercialized as concentrated powders; thus, the different drying procedures applied on fresh* Aloe *gel can modify the molecular structure of constituents and, in particular, may affect acemannan. Regardless of the industrial process (spray drying, freeze drying, refraction window, or radiant zone drying), the molecular weight of acemannan decreased from 49 kDa to 23-26 kDa as did the mannose and the terminal galactose content. And, more important, the degree of acetylation of acemannan was also strongly reduced by 40 to 70%. This reduction not only alters some physicochemical properties but also can affect many of the biological activities attributed to* Aloe vera* [26]. Furthermore, the achieved drying temperature also affects the acemannan constitution: temperatures up to 80 °C promoted an elongation of the acemannan backbone and, at the same time, increased debranching and deacetylation reactions [27]. We have already contributed to elucidate the consequences of heating processes on several* Aloe vera* commercial products: while a spray-dried inner leaf gel powder (ILG) was not cytotoxic to HeLa up to 12 g/l, a belt dried inner leaf powder with a high fibre and polysaccharide content (ILF) was already cytotoxic to HeLa at concentrations of 1-5 g/l, which is even below the recommended concentration for human consumption of 5 g/l [28]. This cytotoxic effect might have been caused by an alteration of the fibres or polysaccharides due to the high temperatures applied during the drying process. Within this context, the aim of this study was to analyse the immunomodulatory effect on human macrophage-like cell line THP-1 induced by* Aloe *gel commercial powders. These powders, used as food supplements, were provided by Mexican facilities of a US enterprise.

## 2. Materials and Methods

### 2.1. Starting Products of* Aloe vera*

The starting products (powders) were provided by Mexican facilities of a US enterprise, which commercializes them as food supplements. On request and in order to obtain samples, the name of the enterprise can be provided. Briefly, the whole leaves were mashed, filtered and pasteurized, filtered with diatomaceous earth, decolourized with activated carbon, filtered (4 *μ*m pore size), and dehydrated by spray drying to a 100× whole leaf powder (WLP), which should be used at 10 g/l (1×). Pasteurized inner leaf fillets were mashed, filtered with diatomaceous earth, decolourized with activated carbon, filtered (4 *μ*m pore size), and dehydrated by spray drying to a 200× inner leaf gel powder (ILG), which mainly contains the soluble acemannan polysaccharide and should be used with 5 g/l (1×). Alternatively, pasteurized inner leaf fillets were belt dried and crushed to a 200× inner leaf fibre powder (ILF), which has a high content of hardly soluble fibres, as well as the soluble acemannan polysaccharide [28].

The samples (WLP, ILG, and ILF) were dissolved by 1x or 5x in culture medium with 50 *μ*g/ml gentamicin (Alexis/Enzo Life Sciences, Farmingdale, USA) and the pH was adjusted with NaOH to pH 7.3±0.1. In order to evaluate the impact of the insoluble fibre content of the ILF-suspension, these samples were further treated to obtain a solution: (a) centrifuged for 20 min at 14,000* g* (Eppendorf 5415D, Hamburg, Germany) (ILF-c) or (b) filtered through a nylon filter with 0.2 *μ*m pore size (ILF-f) or (c) 10 g/l ILF (2×) dissolved in phosphate buffered saline (PBS) and treated for 1 h with 40 g/l activated carbon, filtered (nylon, 0.2 *μ*m pore size), and diluted 1:1 with culture medium supplemented with 20% FBS (Biowest, Kansas City, USA) (ILF-ac).

### 2.2. Cell Lines and Culture Conditions

The cell lines THP-1 and Caco-2 (#TIB-202 and #HTB-37, both ATCC, Manassas, USA) were grown at 37 °C, 4% CO_2_, and 95% RH in DMEM/F12 (Caisson, Smithfield, USA) and DMEM (ATCC), respectively, both supplemented with 10% FBS.

For the tests, 3*∗*10^5^ THP-1 or 6*∗*10^4^ Caco-2 were seeded in 1 ml culture medium (when necessary on cover slides) in 12-well multititre plates. For cytotoxicity studies, the human colon line Caco-2 was incubated for 3 d for attachment and to achieve a high confluence of > 90%, before the medium was changed for sample-containing medium. The cells were then grown for 4 h or 24 h prior to analysis. For immunomodulatory studies, the monocyte cell line THP-1 was differentiated to macrophage-like cells by adding 1 *μ*M phorbol-12-myristate-13-acetate (PMA; Sigma-Aldrich, St. Louis, USA) and dissolved in PBS, to the culture medium, followed by incubation for 3 d; the differentiation was checked by observing cell adherence before the medium was changed for sample-containing medium.

### 2.3. Cytotoxicity: Caco-2

Metabolic activity was determined qualitatively by neutral red uptake (NRU) and quantified using the WST-test. The sample-containing medium was removed, the cells were washed with PBS and fresh culture medium containing 20 *μ*l/ml WST-1 (Clontech, Mountain View, USA) and 20 *μ*l/ml 0.33% neutral red solution (Santa Cruz, Santa Cruz, USA), respectively, were added, and the cells were incubated for further 4 h. For the NRU-test, cover slides were washed once with PBS and observed under the microscope (Axioskop 40 FL, Zeiss, Oberkochen, Germany). For the WST-test, 900 *μ*l medium was removed and centrifuged (1 min, 8,000* g*) and the absorbance was measured at *λ* = 440 nm and *λ* = 690 nm (as background) (Mecasys Optizen-Pop, Daejeon, South Korea).

### 2.4. Immunomodulation: THP-1

After THP-1 monocytes were differentiated to macrophage-like cells and exposed for 1 h to the different samples (medium change), the cells were challenged with 1 *μ*g/ml LPS (L4291, Sigma-Aldrich). After 3 h and 24 h, respectively, 400 *μ*l culture medium was removed, centrifuged (15 min, 1,500* g*), and stored at −80 °C until further analysis. At the same time, 10 *μ*l trypan blue was added to the residual medium and cell viability was analysed by microscopy.

### 2.5. Cytokine Release: TNF*α*, IL-1*β*, IL-10

The centrifuged and stored supernatants were analysed by ELISA for the proinflammatory cytokines TNF*α* and IL-1*β* as well as for the anti-inflammatory cytokine IL-10 (Elabscience, Wuhan, China). Briefly, up to 100 *μ*l supernatant was added into, with the specific antibody coated, wells of a 96-well microtitre plate, incubated, supernatant removed, specific biotinylated antibody added, incubated, washed, avidin-horseradish peroxidase conjugate added, incubated, washed, reaction solution added (not stated in the manual, probably 3,3′,5,5′-tetramethylbenzidine), acidic stop solution added, and the absorption at *λ* = 450 nm was determined for each well. The following samples were analysed: blank (medium + sample), mock control (diff. THP-1), negative control (diff. THP-1 + sample), positive control (diff. THP-1 + LPS), and test (diff. THP-1 + sample + LPS).

### 2.6. Statistical Analysis

Values are expressed as means ± 1.96 *∗* standard error of mean (1.96*∗*SEM). Differences between groups were determined by one-way analysis of variance (ANOVA) using the program Origin 5.0.

## 3. Results

### 3.1. Cytotoxicity

**Figure 1:**
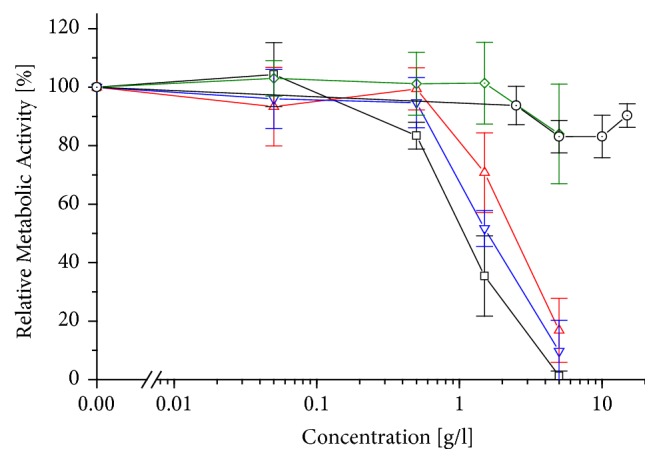
Metabolic activity (WST-1) of Caco-2 after 4 h exposure to differently treated* Aloe*-starting products and estimation of the cytotoxic concentration CC_50_: ILG (dotted circle), ILF (square), ILF-c (up triangle), ILF-f (down triangle), and ILF-ac (dotted diamond). Values are expressed as mean ± 1.96*∗*SEM (n = 4).

Previously, cytotoxic effects of ILF on HeLa cells were reported [28]; therefore, in this study, cells of first contact with foodstuff, the intestinal cell line Caco-2, were analysed.* Aloe* gel extract with a high fibre content (ILF) already exhibited a cytotoxic effect with a CC_50_ = 1.0 g/l after 4 h of exposure (Figure 1). It should be highlighted that this value is below the recommended concentration of 5 g/l for foodstuff.* Aloe* gel extract with a reduced content of fibres, that is, ILG, was not cytotoxic up to 15 g/l (Figure 1). Neither by filtration nor by centrifugation the cytotoxicity of the ILF samples could be reduced; cytotoxic effects with CC_50_ = 1.5 g/l for ILF-f and CC_50_ = 2.5 g/l for ILF-c (Figure 1) could still be found at concentrations below the recommended 5 g/l for ILF. Only a treatment of ILF with activated carbon (ILF-ac) reduced its cytotoxic effect considerably to CC_50_ > 5.0 g/l (Figure 1).

Overall, these results indicate that heated* Aloe vera* samples, when containing fibres (*i.e.*, ILF), are cytotoxic and that this cytotoxic principle can be remediated by a treatment with activated carbon.

### 3.2. THP-1 Differentiation

**Figure 2:**
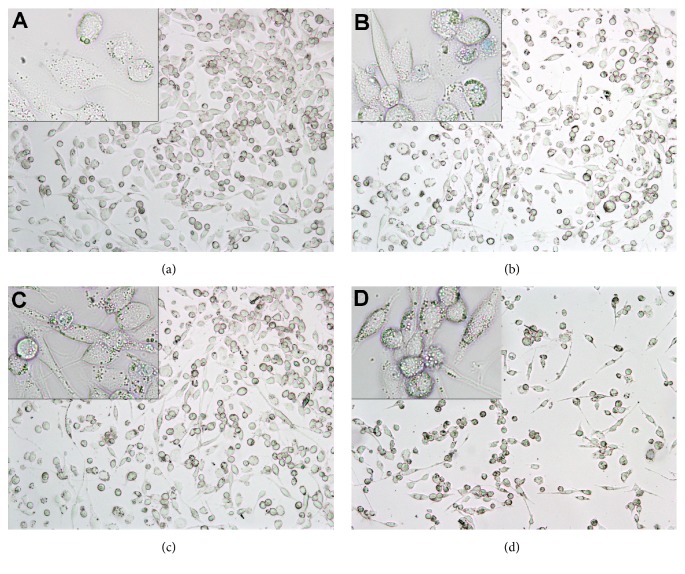
Trypan blue stained cells with compromised membrane are blue: differentiated THP-1 (a). Differentiated THP-1 challenged with 1 *μ*g/ml LPS and additionally exposed to 2 g/l (0.2×) WLP (b), 1 g/l (0.2×) ILG (c), and 25 mg/l (0.005×) ILF (d), respectively.

**Figure 3:**
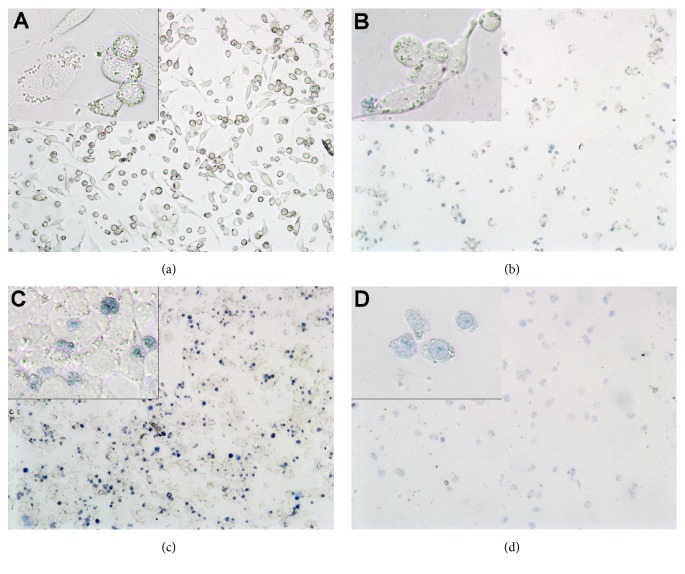
Trypan blue stained cells with compromised membrane are blue: differentiated THP-1 cells after being challenged for 24 h with 1 *μ*g/ml LPS (a). Cells additionally exposed to 10 g/l (1×) WLP (b), 5 g/l (1x) ILG (c), and 5 g/l (1×) ILF (d), respectively.

Cell confluence affects the quantity of produced cytokines; thus, cells were observed microscopically for each sample to (a) prove the correct differentiation of THP-1 to macrophage-like cells and (b) estimate the confluence. After treatment with 1 *μ*M PMA for 3 d, THP-1 monocytes successfully differentiated into macrophage-like cells (Figure 2(a)). While THP-1 monocytes grew in suspension and thus were round, differentiated THP-1 cells were bigger and oval, grew adherent, and presented more granules. Those cells were subsequently treated with* Aloe* supplements and then challenged with 1 *μ*g/ml LPS (Figure 3(a)). At low concentrations (0.2× for WLP and ILG, and 0.005× for ILF) no alterations either in cell morphology or in staining behaviour by trypan blue could be observed (Figures 2(b)–2(d)); that is, the cells were not inhibited by a cytotoxic effect of the sample itself.

**Figure 4:**
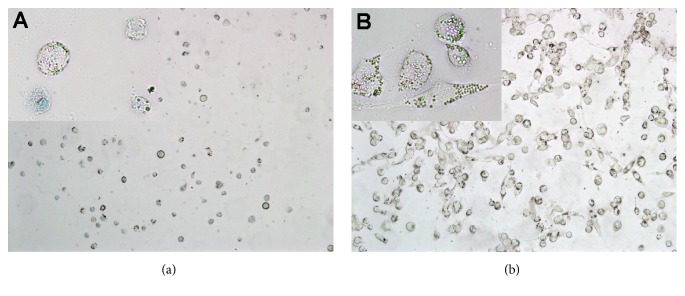
Trypan blue stained cells with compromised membrane are blue: differentiated THP-1 cells challenged with 1 *μ*g/ml LPS and additionally exposed to 1 g/l (0.2×) ILF (a) and 1 g/l (0.2×) ILF-ac, respectively.

The opposite effect could be seen at higher concentrations: at 1x (10 g/l) WLP, confluence was reduced and the cells were stained bluer and rounded (dead) (Figure 3(b)). At 1× (5 g/l) ILF, the number of cells was clearly reduced and almost all cells were stained by trypan blue (Figure 3(d)). This result could be expected, as ILF has a CC_50_ = 1.0 g/l for HeLa [28] or Caco-2 (Figure 1). Even at 0.2× ILF, the cell number was reduced and many cells were rounded and stained blue (Figure 4(a)); this indicates that differentiated THP-1 are far more sensitive to ILF than HeLa or Caco-2. However, when this sample was treated with activated carbon (ILF-ac), the same concentration was not toxic to differentiated THP-1 (Figure 4(b)). On the other hand, 1× (5 g/l) ILG did not reduce confluence, but several cells were stained blue (dead) (Figure 3(c)), which coincides with the low cytotoxicity against HeLa [29] and Caco-2 (Figure 1).

### 3.3. Immunomodulation

**Figure 5:**
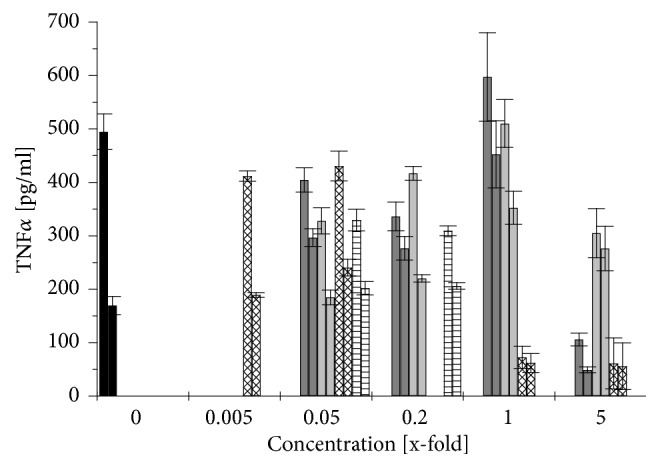
Changes in TNF*α* release after 24 h from differentiated THP-1 cells induced by a treatment with different* Aloe vera* products and LPS stimulation (left columns) compared to that without LPS stimulation (right columns). 1x refers to the recommended concentration for human consumption: control without* Aloe vera* treatment (black), WLP (grey), ILG (light grey), ILF (rhombus patterned), and ILF-ac (horizontally lined) columns. Values are expressed as mean ± 1.96*∗*SEM (n = 3).

**Figure 6:**
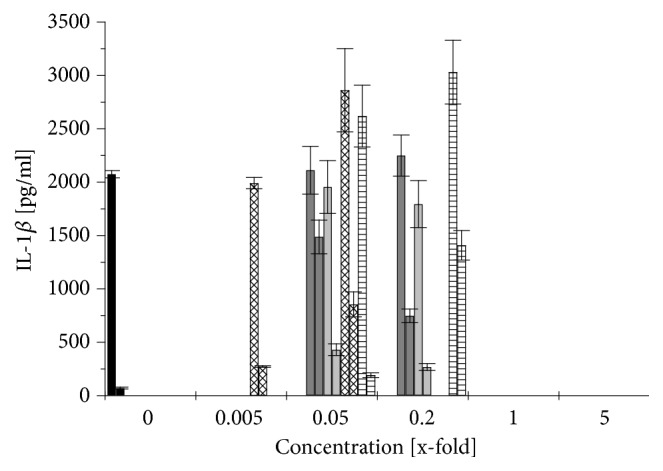
Changes in IL-1*β* release after 24 h from differentiated THP-1 cells induced by a treatment with different* Aloe vera* products and LPS stimulation (left columns) compared to that without LPS stimulation (right columns). 1x refers to the recommended concentration for human consumption: control without* Aloe vera* treatment (black), WLP (grey), ILG (light grey), ILF (rhombus patterned), and ILF-ac (horizontally lined) columns. Values are expressed as mean ± 1.96*∗*SEM (n = 3).

**Figure 7:**
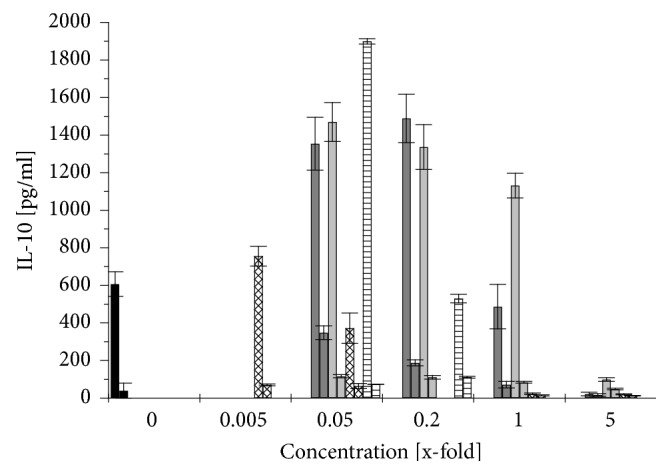
Changes in IL-10 release after 24 h from differentiated THP-1 cells induced by a treatment with different* Aloe vera* products and LPS stimulation (left columns) compared to that without LPS stimulation (right columns). 1x refers to the recommended concentration for human consumption: control without* Aloe vera* treatment (black), WLP (grey), ILG (light grey), ILF (rhombus patterned), and ILF-ac (horizontally lined) columns. Values are expressed as mean ± 1.96*∗*SEM (n = 3).

The immunomodulatory effect of different* Aloe vera* products was determined using differentiated THP-1 cells. The cells were first exposed to the different* Aloe vera* products for 1 h and then challenged with 1 *μ*g/ml LPS. The cytokine release was measured after 3 and 24 h of exposure to the LPS. Figures 5, 6, and 7 show the changes of cytokine release caused by an* Aloe*-treatment respective to the cytokine release induced by LPS treatment alone. Although no clear changes could be observed after 3 h, the effects were more obvious after 24 h: stimulation by LPS alone increased the release of the proinflammatory cytokines TNF*α* by 300% and IL-1*β* by even 29 times while the anti-inflammatory cytokine IL-10 increased up to 15 times compared to not-challenged THP-1 (Figures 5–7, black columns).

A treatment of differentiated THP-1 with low concentrations of WLP (0.05× and 0.2×) increased the release of TNF*α* by 60 to 75%, of IL-1*β* even by 10 to 20 times, and of IL-10 by 5 to 9 times (Figures 5–7,* right *grey columns). But when the differentiated THP-1 were challenged with LPS, then this WLP-treatment reduced TNF*α*-release slightly by 20 to 33%, IL-1*β*-release remained stable, and the anti-inflammatory cytokine IL-10 increased by 2 to 2.5 times (Figures 5–7,* left *grey columns). WLP at 1× reduced cell density of differentiated and challenged THP-1 (Figure 3(b)); nevertheless, the TNF*α*-release still increased by 20%, while WLP at 5× was cytotoxic and inhibited cytokine release (Figure 5,* left *grey columns).

Generally, at nontoxic concentrations, WLP stimulated the release of the proinflammatory cytokines TNF*α* and IL-1*β* from differentiated THP-1, while on challenged THP-1 the proinflammatory cytokine release remained stable or was slightly reduced compared to untreated THP-1. The anti-inflammatory cytokine IL-10 increased in both cases: challenged and not-challenged THP-1. Only at a nonphysiological and nontoxic concentration of 1× WLP, the TNF*α*-release increased even of not-challenged THP-1.

ILG caused a similar behaviour on differentiated THP-1 like WLP, although less pronounced: at low concentrations (up to 0.2×), the proinflammatory cytokines increased by 10 to 30% for TNF*α* and by 4 to 6 times for IL-1*β*, while the anti-inflammatory cytokine IL-10 increased but remained over all at a low level (Figures 5–7,* right* light grey columns). Again, when the differentiated THP-1 were challenged with LPS, up to 0.2× ILG reduced the liberation of proinflammatory cytokines: TNF*α* by 15 to 35% and IL-1*β* slightly by 5 to 15%; on the other side, the anti-inflammatory cytokine IL-10 increased by 2.2 to 2.4 times (Figures 5–7,* left* light grey columns). At 1×, ILG increased TNF*α*-release of differentiated THP-1 by 2 times (Figure 5,* left* light grey columns).

All in all, ILG has a similar immunomodulatory effect like WLP: at low concentrations, ILG stimulated the release of proinflammatory cytokines from not-challenged THP-1, while on LPS challenged THP-1 the liberation of these cytokines was (slightly) reduced. The effect on the anti-inflammatory cytokine was inverse: for not-challenged THP-1 the values increased but remained on a low level and on LPS challenged THP-1 IL-10-release increased clearly.

As the ILF sample was already cytotoxic at a concentration > 0.2× (Figure 4(a)), at this concentration, no cytokine release could be observed (Figures 5–7, rhombus patterned columns). At low concentrations (up to 0.05×), ILF itself stimulated unchallenged but differentiated THP-1 to increase the release of the proinflammatory cytokine TNF*α* by up to 40%, while the liberation of IL-1*β* increased up to 12 times (Figures 5 and 6,* right* rhombus patterned columns). Under this conditions, the liberation of the anti-inflammatory cytokine IL-10 increased by 1.7 times (Figure 7,* right* rhombus patterned columns). When differentiated THP-1 were challenged with LPS the effect caused by ILF depended strongly on the used concentration: only at 0.005× a slight anti-inflammatory effect could be observed (IL-10 increased by 23%), but already at 0.05× this effect was inverted: IL-1*β* increased by 35% and IL-10 decreased by 40% (Figures 6 and 7,* left* rhombus patterned columns).

Finally, when the fibre containing sample was treated with activated carbon (ILF-ac) things changed: first of all, the sample was less cytotoxic (Figure 4) but at low concentrations (0.05×) the capacity to stimulate the IL-10 release to similar values to those observed for ILG was also restored (Figure 7,* left* horizontal lined columns). Surprisingly, at the still noncytotoxic concentration of 0.2×, ILF-ac already reduced IL-10 release to approximately 85% (Figure 7,* right* horizontal lined columns). This indicates that the ILF sample could be detoxified by an activated carbon treatment and was gaining some anti-inflammatory properties.

## 4. Discussion

In a previous work it was found that the inner leaf gel of* Aloe vera* (ILG) was not toxic for the human cervical cell line HeLa [29], whereas the inner leaf gel with a high fibre content (ILF) was cytotoxic for HeLa with a CC_50_ = 1 g/l, which is even below the recommended concentration for human consumption [28]. In this study, those previous results could be confirmed with the more relevant human intestinal cell line Caco-2, cells which may be exposed to high concentrations of* Aloe vera* products after their ingestion. Both samples, ILG and ILF, were obtained from the same source (*Aloe vera* inner leaf gel); however, they are prepared in a very different manner. On one hand, the gel with its soluble polysaccharides was extruded for the ILG sample (thus has a low insoluble fibre content), treated with activated carbon and spray-dried at temperatures of about 60 °C. On the other hand, the gel containing all the fibre and polysaccharide content was only dehydrated at 70-85 °C for the ILF sample and hence not decolourized with activated carbon (a more detailed description of the sample preparation is given in [28]). Since both samples arise from the same source, the different applied treatments should explain the different cytotoxic effects. Now, the cytotoxic effect of ILF on Caco-2 (CC_50_ = 1 g/l) could not readily be reduced by centrifugation or filtration (Figure 1), but, by a treatment with activated carbon, the CC_50_ increased to > 5.0 g/l. These results indicate that the polysaccharides themselves are not cytotoxic. This coincides with the fact that acemannan isolated from* A. vera* is not cytotoxic to human gingival fibroblasts; in fact, at concentrations of 1 and 10 mg/ml, it even stimulated cell growth [24]. The acemannan polymer can undergo a deacetylation process when exposed to temperatures above 70 °C [27, 30, 31]; but even deacetylated acemannan does not exhibit a cytotoxic effect [32].

The human monocyte cell line THP-1 is much more sensitive to* Aloe*-starting products than the intestinal line Caco-2: WLP and ILG were cytotoxic at 1× (*i.e.*, 10 g/l and 5 g/l, respectively) and ILF was cytotoxic even at 0.2× (1 g/l; Figure 3); only at concentrations of 0.2× WLP, ILG, or 0.005× ILF did the cells grow normally (Figure 2). This coincides with results that 10%* Aloe* gel (0.1×) reduced cell growth of THP-1 by only 20% [13]. However, under physiological conditions, it cannot be expected that blood cells like THP-1 would be exposed to such high concentrations as intestinal cells. Thus, relevant immunomodulatory effects should be expected at these or even lower concentrations.

There are three possible stimuli that induce an inflammatory response: infection, tissue injury or stress, and metabolic malfunction. For instance, the PAMP LPS is recognized by TLR4/MyD88, which activates the NF-*κ*B signalling cascade and induces the synthesis of pro-IL-1*β* and NLRP3. Activation of the NLRP3-inflammasome can occur by extracellular ATP, recognized by the P2X7-receptor, leading to an efflux of cytosolic K^+^ through pannexin-1: then NLRP3 associate with ASC and activate pro-caspase-1, which in turn activates the proinflammatory cytokine IL-1*β* by limited proteolysis [33, 34]. As part of the inflammatory response, the release of IL-1*β* from infiltrating neutrophils induces the activation of several cell types such as macrophages, T-lymphocytes, epithelial, and endothelial cells and enhances the expression of further proinflammatory cytokines like IL-6 and TNF*α* [13, 35]. However, a prolonged synthesis of proinflammatory cytokines contributes to a local or systemic inflammation, which may further develop into chronic diseases [36]; therefore, also their downregulation is required.

The polysaccharide fraction from* Aloe* inner gel, mainly acemannan, has been demonstrated to be immunomodulatory to monocytes and macrophages in several studies [13, 21–23]. In agreement with the latter results, no significant changes in the liberation of proinflammatory cytokines (TNF*α*, IL-1*β*) were observed when differentiated and with LPS challenged THP-1 were exposed to samples with a low fibre and polysaccharide content (WLP, ILG) at low concentrations (up to 0.2×): the release of TNF*α* was reduced by 15 to 35% and of IL-1*β* did not change (Figures 5 and 6,* left* grey and light grey columns). Budai* et al.* (2013) found that a commercial* Aloe* solution at 10% (which would correspond to 0.1× ILG) decreased TNF*α* and IL-1*β* release by 60% and 40%, respectively, when differentiated THP-1 cells were challenged by LPS [13]. This may be explained by a downregulation of LPS-induced expression of NLRP3, pro-caspase 1, and P2X7R by the* Aloe* extract. However, here we report for the first time that, under these conditions, the anti-inflammatory cytokine IL-10 increased (Figure 7,* left* grey and light grey columns), which would support a general anti-inflammatory effect. This anti-inflammatory effect could also be demonstrated in Wistar rats with induced peritoneal* Staphylococcus aureus* infection:* Aloe* gel extract alone increased the liberation of IL-1*β* and, as expected, infected animals exhibited increased IL-1*β*-levels. However, when the infected rats were treated with* Aloe* gel extract, the liberation of IL-1*β* decreased to normal values again [37].

The opposite effect was exhibited by the sample with the high fibre and polysaccharide content (ILF) at 0.05×: while the proinflammatory cytokine IL-1*β* increased slightly by 35% (Figure 6,* left *rhombus patterned column), the anti-inflammatory cytokine IL-10 decreased by 40% (Figure 7,* left *rhombus patterned column). Similarly, Pugh* et al.* (2001) noted an increase of proinflammatory cytokine release (TNF*α*, IL-1*β*), when LPS challenged THP-1 cells were exposed to the > 500-kDa polysaccharide fraction [23]. The mouse macrophage cell line RAW264.7 also increased proinflammatory cytokine release when in contact with acemannan [21] or with a purified polysaccharide fraction of 5-400 kDa [22]. This would indicate that, apart from the cytotoxic effect of ILF samples, the immunomodulatory effects of ILF are not as desirable as those from ILG samples. Interestingly, when ILF at this concentration was treated with activated carbon (ILF-ac), not only was the cytotoxicity reduced but also the anti-inflammatory cytokine IL-10 increased by 3.1 times (Figure 7,* left* horizontal lined column) even surpassing the effects of ILG.

*Aloe* inner leaf gel products, treated with activated carbon (in order to reduce the aloin content) and spry dried at relatively low temperatures, exhibited more advantageous properties (low cytotoxicity, anti-inflammatory effect) than the recently introduced* Aloe* inner leaf gel with a high fibre content. Since these products were not treated with activated carbon, a cytotoxic principle is not removed and, by having high fibre content, may promote proinflammatory effects.

## Data Availability

The raw data (from the photometer) as well as (also additional) photos used to support the findings of this study are available from the corresponding author upon request.

## Abbreviations

ASC: Apoptosis associated Speck-like protein containing Card domain
ATCC: American Type Culture Collection
DMEM: Dulbecco's Modified Eagle Medium
FBS: Fetal Bovine Serum
FDA: Food and Drug Administration (USA)
IASC: International Aloe Science Council
ILF: * Aloe* inner leaf fibre powder
ILG: * Aloe* inner leaf gel powder
LPS: Lipopolysaccharide
NLRP3: Nucleotide-binding oligomerization domain, Leucine-rich repeat, and Pyrin domain-containing 3
NRU: Neutral red uptake
PAMP: Pathogen-associated molecular pattern
PBS: Phosphate buffered saline
RH: Relative humidity
RT: Room temperature
TLR: Toll-like receptor
WLP: * Aloe* whole leaf powder
WST: Water soluble tetrazolium
